# A Qualitative Study of Collaborative Food Programs: Insights from a FQHC–University Partnership During COVID-19

**DOI:** 10.3390/nu17111856

**Published:** 2025-05-29

**Authors:** Miranda Kim, Christine K. Thang, Lauren Imai, Marius Corwin, Mopelola A. Adeyemo, Catherine Imbery, Shanika Boyce, Cambria L. Garell, Wendelin M. Slusser, Alma D. Guerrero

**Affiliations:** 1 Semel Healthy Campus Initiative Center, UCLA, Los Angeles, CA 90095, USA; 2 Department of Pediatrics, UCLA David Geffen School of Medicine, Los Angeles, CA 90095, USAaguerrero@mednet.ucla.edu (A.D.G.); 3 Department of Community Health Sciences, UCLA Fielding School of Public Health, Los Angeles, CA 90095, USA; 4 Department of Medicine, UCLA David Geffen School of Medicine, Los Angeles, CA 90095, USA; 5 Department of Pediatrics, Charles R. Drew University of Medicine and Science, Los Angeles, CA 90095, USA; shanikaboyce@cdrewu.edu

**Keywords:** food insecurity, COVID-19, food is medicine, community health, food pharmacy, produce prescription, nutrition, prepared meal program, food program, emergency response

## Abstract

Background/Objectives: This study aims to fill gaps in the existing literature through a qualitative evaluation of stakeholders involved in Food Is Medicine (FIM) programs. The primary objective was to examine the structural components, implementation process, and perceived impact of the University of California Los Angeles (UCLA) and Venice Family Clinic (VFC) Emergency Prepared Meal Program (UCLA-VFC Food Program), developed as a collaboration between a university and Federally Qualified Health Center (FQHC) during a period of community crisis. A secondary objective was to compare this program with three other FIM initiatives, identifying convergences and divergences in design and effectiveness. Methods: The methodology involved semi-structured interviews conducted with stakeholders across all four programs. Participants were recruited based on their direct involvement with program ideation, design, or implementation. Interviews were conducted online between July and September 2022, with 11 stakeholders. A thematic analysis was applied to the transcribed responses using an inductive thematic analysis. Results: Key findings highlighted four recurrent themes: (1) the critical role of leadership and a collaborative culture; (2) the importance of community partnerships and health education; (3) challenges related to logistics, funding, and sustainability; and (4) the need for assessment and evaluation. These findings provide valuable insight into the design of future FIM interventions, particularly those embedded in healthcare settings. Conclusions: In conclusion, this study offers preliminary evidence supporting the unique potential of university–community partnerships to address food insecurity. Unlike previous research that emphasized clinical outcomes, our findings provide a contextualized understanding of programmatic implementation. While further quantitative evaluation is necessary, this work lays the groundwork for a collaborative model between various entities including universities, healthcare systems, clinics, and community health/food services aimed at addressing social determinants of health.

## 1. Introduction

Food insecurity is one of the social determinants of health associated with negative health outcomes [1]. The United States Department of Agriculture defines food insecurity as a household-level economic and social condition of limited or uncertain access to adequate food [2]. In 2020, 10.5% of US households experienced food insecurity, with a higher prevalence of 28.6% among low-income households [3]. Rising unemployment rates and income loss as a result of the COVID-19 pandemic worsened the existing food insecurity problem. Between April and July 2020, 41.6% of households below 300% of the federal poverty level (FPL) experienced food insecurity. Moreover, between April and December 2020, 34% of households across all socioeconomic levels experienced food insecurity [4].

Addressing food insecurity and improving access to healthy food choices have the potential to improve overall health. A Food Is Medicine (FIM) framework can help conceptualize various ways to address food insecurity while improving health. Such a framework encompasses a myriad of food and nutrition program designs. These range from more intensive—with specifically tailored meals to manage chronic conditions—to less intensive strategies to promote population-level health and food security [5]. Many FIM programs are universally bound by a common effort to address food assistance and increase access to nutritious foods. They are also uniquely positioned to integrate nutrition, medicine, and community health efforts. A systematic review and meta-analysis found that healthcare-based FIM programs in particular may help improve community access to healthy foods including fruit and vegetables [6]. In addition, successful programs have shown promise in improving health measures such as decreasing blood sugar concentrations, lowering blood pressures, promoting weight loss for type 2 diabetics, and decreasing healthcare spending [7,8,9,10,11].

Prepared meal programs, such as the partnership between the University of California Los Angeles (UCLA) and Venice Family Clinic (VFC) in the UCLA-VFC Emergency Prepared Meal Program (UCLA-VFC Food Program), serve as an example of a FIM approach [12]. A prepared meal program is distinct from food pantries in that a prepared meal program goes a step beyond providing ingredients and distributes pre-made meals. This approach lies closer to the treatment end of the prevention-to-treatment spectrum described by the FIM framework and may be particularly useful in closing the hunger gap in vulnerable communities by overcoming barriers to the utilization of community resources. Importantly, this approach can serve as a starting point for a robust health education intervention aimed at equipping patients to make and consume low-cost nutritious meals [5].

Nationwide, similar prepared meal programs exist, including meal delivery and produce-prescription programs [7,9]. Such programs aim at overcoming barriers to access by delivering meals directly to patients and partnering with local farmers’ markets, respectively. However, there is little discussion surrounding how FIM programs compare in terms of their infrastructure, logistics, and impact on their respective communities. The current literature also lacks insights on how to set up a framework or replicable protocol for meal provision to food insecure communities in an FQHC clinical setting. This paper aims to fill in these gaps through a qualitative analysis of interviews with various program stakeholders. Our primary goal was to examine the components of the UCLA-VFC Food Program as an approach to address food insecurity in an FQHC setting during a community crisis. A secondary goal was to examine other FIM efforts and identify similarities and differences across programs. We discuss the qualitative findings to inform future community healthcare partnerships and food insecurity interventions while contributing to the dialogue at the intersection of healthcare, nutrition, and community wellbeing.

## 2. Materials and Methods

### 2.1. UCLA-VFC Food Program Setting and Context

The COVID-19 pandemic impacted universities and health systems in unique ways, creating opportunities for collaboration. As undergraduate students moved out of the dorms in March 2020, UCLA Dining Services reduced their operations and university staff faced a potential loss of employment. The Venice Family Clinic (VFC), an FQHC affiliated with UCLA that serves over 45,000 low-income families annually, sought to continue and expand patient services in light of the various job losses, school closures, and financial strain affecting families cared for by the clinic [13]. Leveraging UCLA Chancellor Gene Block’s commitment to maintain employment for dining staff, UCLA partnered with the VFC to prepare and deliver meals to patients and their families, thereby maintaining university staff employment and addressing increased community needs.

Facilitated by UCLA’s Semel Healthy Campus Initiative Center (Semel HCI Center), the UCLA-VFC Food Program leveraged existing infrastructure and staffing to provide over 525,000 prepared meals to approximately 41,600 people over 12 months (August 2020–July 2021) [14]. The VFC secured funding for the cost of food, while UCLA Dining provided the labor to prepare meals for the clinic’s patients and families. Initially, the program prioritized medically vulnerable patients and later expanded its reach to all patients and their households once logistics were better established. Refrigerated trucks delivered meals from UCLA to VFC sites four days a week. The meals followed recipes from the Bruin Plate Cookbook, a collection of recipes developed by UCLA Dining, which ensured each meal contained standard portions of protein, vegetables, and starch (five ounces each) [15,16]. The UCLA-VFC Food Program scaled up recipes to serve larger families (e.g., Chickpea Panisse for six people) and downsized for smaller groups (e.g., Red Lentil Stew for two).

### 2.2. Program Participants’ Characteristics and Clinical Data

In partnership with the VFC Information Technology (IT) team, a subset of individuals was identified for whom there were electronic health record (EHR) data one year before, during, and after participation in the UCLA-VFC Food Program. The IT team extracted data from participants’ EHRs including height, weight, body mass index (BMI), blood pressure (BP), and biomarker labs. These variables are often used to monitor weight-related health risks. The data were retrieved from one year before, during, and one year after participation in the UCLA-VFC Food Program.

### 2.3. Program Stakeholder Interviews

To explore the development, implementation, and impact of the UCLA-VFC Food Program and similar food programs, a qualitative study was conducted through in-depth, semi-structured interviews with stakeholders from the UCLA-VFC Food Program and three other FIM programs. These food programs were selected based on their integration of academic and community partnerships.

We recruited participants between July and September 2022 through email outreach, word-of-mouth (snowball) sampling, and a literature review of related publications. The study team identified potential participants through a literature review with the PubMed database using the keywords: “food pharmacy”, “food insecurity”, “COVID-19 food insecurity”, and “COVID-19 meal programs”, and contacted the authors listed on these papers. There was outreach to individuals who led and operationalized food programs, including program administrators, directors, and managers. Email invitations were sent, and initial contacts were asked to refer other individuals for this study. Prospective participants were invited to schedule Zoom interviews (Zoom Video Communications, Inc., San Jose, CA, USA). Participation was voluntary, and participants received a $30 electronic gift card for their time. Verbal consent was obtained before audio-recording interviews. Transcription was completed using Otter.ai, an artificial intelligence transcription tool. The remaining interviews were documented through written notes as requested by those interviewees who declined audio-recording.

A semi-structured interview guide consisting of 15 questions was developed based on the literature review and input from content experts and the research team. Questions focused on program successes (e.g., “What do you see as the major successes of the program?”), challenges (e.g., “What were some of the challenges to running the program?”), logistics, lessons learned, and future directions. The interview guide also explored participants’ involvement and lessons learned.

Grounded theory and an inductive thematic analysis guided the coding and identification of key themes from the interviews. One research team member developed a preliminary codebook based on topics and concepts from the initial interviews. In conjunction with the research team, this preliminary code book was used and built upon to determine whether any new information was gained as more of the interviews unfolded. The last interview was completed based on whether any new topics or concepts emerged in discussion with the research team. Two members of the research team (authors MC and MK) then reviewed the de-identified interview transcripts, and developed codes using a consensus-seeking, iterative discussion. The codes were used to develop the main themes. Recurrent themes were identified and compared by the initial two reviewers. The final themes were identified through discussion, refinement, and reconciliation of any differences in coding by the initial two reviewers. After this process, a third reviewer independently reviewed the transcripts and set of themes. All of the themes identified by the third reviewer aligned with those identified by the two initial reviewers. Representative interview quotes were selected to illustrate the major themes. Data collection was approved by the UCLA Institutional Review Board (UCLA IRB#22-000597).

## 3. Results

### 3.1. UCLA-VFC Food Program

The UCLA-VFC Food Program delivered approximately 525,000 prepared meals to approximately 41,600 individuals from VFC over approximately 12 months, from August 2020 to July 2021 [14]. Demographic characteristics and clinical trends of the subset of 6,088 program participants provided by the VFC IT team are listed below. Table 1 provides a demographic summary of program participants and Table 2 shows their clinical data. Approximately one-third of the meals distributed were to children and youth (age < 20 years). Over half of the meals were distributed to individuals who identified as females (63%) and who were Hispanic white (76%). Most meals were distributed to adults and children who were publicly insured and not homeless. Regarding clinical measures, Table 2 shows the average BMI, BP, and weight before, during, and a year after participating in the UCLA-VFC Food Program. These are descriptive averages at the three time points.

### 3.2. Program Details and Interview Findings

Eleven interviews were conducted, including with representatives from the UCLA-VFC Food Program and three other food programs across the country from July to September 2022. Interviews averaged 37 min in length. To maintain anonymity among interviewees, food programs outside of the UCLA-VFC Food Program—including produce prescription and other FIM initiatives—are referred to in general terms. The University-Affiliated Program is a program initiated and run by a university, the Public Health Program is led by a county department of public health, and the Nonprofit Program is a collaborative organization with several community partners. Collectively, we refer to these three programs as Non-UCLA-VFC Programs in this paper. Interviews with representatives from the UCLA-VFC Food Program, including UCLA Dining leaders, UCLA Dining staff, VFC healthcare providers, and VFC administrators, are collectively referred to as part of the UCLA-VFC Food Program. Table 3 details characteristics of the interviewees and their respective programs.

The thematic analysis of the 11 interview transcripts resulted in four key themes with 12 subthemes. These findings summarize critical insights into the origins, development, and implementation of food programs. The four key themes centered on (1) leadership and culture; (2) partnerships and health education; (3) logistics, funding, and sustainability; and (4) assessment and evaluation. Table 4 summarizes the key themes, subthemes, and representative quotes.

#### 3.2.1. Theme 1: Leadership and Culture

The UCLA-VFC Food Program interviewees emphasized the importance of having visionary leadership with an unwavering commitment to improve the health and wellbeing of local communities. Their ability to cast vision with enthusiasm promoted a positive and service-oriented culture. Prior experience with cross-campus collaborations also positioned leaders to harness the skills and resources of the campus and community partners to develop the emergency meal program during the COVID-19 pandemic. With existing infrastructure and a collaborative culture, UCLA and VFC mobilized community resources quickly and efficiently to respond in real-time. Program interviewees indicated that their leaders played a crucial role in harnessing philanthropy, inspiring collaboration, and leveraging creative problem-solving. Providers and university administrators alike shared a common goal to serve the community and were guided by a FIM ideology. Interviewees from the Non-UCLA-VFC Programs shared similar comments about the importance of leadership and a collaborative culture.

#### 3.2.2. Theme 2: Partnerships and Health Education

Strategic partnerships and health education underscored the value of collaborative and educational approaches. All interviewees emphasized building relationships with stakeholders within and outside of their organizations, and the role of education in enhancing community engagement and program effectiveness. Such collaborations expanded the reach of these programs beyond clinic patients to impact broader neighborhoods and communities.

All interviews emphasized how health educators played a vital role in integrating nutrition and health education for the recipients of their services. Education and training efforts involved multiple partners including nurses, social workers, and other healthcare and administrative staff. Programs provided immediate food assistance while equipping patients to make informed choices about the foods they prepare and consume. The University-Affiliated Program noted that this education enabled healthcare staff and providers to support patients’ behavioral changes beyond simply following recipes. Education provided patients with the rationale of “why” behind their healthful choices, while the foods or meals offered examples as to “what” healthy options look like and “how” they can be prepared. The prepared meals also helped to overcome barriers such as the time needed to purchase and prepare the foods, particularly for those working or caretaking for their family. The repeated theme of implementing an educational component to bolster the impact of food programs was shared across the Non-UCLA-VFC Programs. In contrast, the UCLA-VFC Food Program—implemented as a rapid response during the pandemic—lacked a formal health education component, which was more established in the other programs. Nevertheless, the UCLA-VFC Food Program recognized the importance of providing health education. This was reflected in interviewees’ descriptions of informal conversations they had with patients about meals and nutrition in their daily interactions.

#### 3.2.3. Theme 3: Logistics, Funding, and Sustainability

Interviewees acknowledged the logistical and financial considerations influencing the sustainability and reach of their programs. All interviewees discussed the challenges of securing funding, managing costs, and optimizing operational processes to efficiently deliver services. The UCLA-VFC Food Program relied on initial philanthropic donations to fund food costs, while UCLA Dining staff contributed labor to prepare the meals. The Non-UCLA-VFC Programs were funded similarly through philanthropy and grants. All interviewees emphasized the importance of collaborations with food pantries, which provided ingredients, organizational infrastructure, and staff to help with distribution. Interviewees also acknowledged the role of food donations and volunteer staffing (i.e., local students and community residents) in helping them overcome shortages of resources and personnel.

#### 3.2.4. Theme 4: Assessment and Evaluation

Since the UCLA-VFC Food Program lacked a formal needs assessment, interviewees from this program stressed the importance of implementing an evaluation to guide ongoing improvement and measure impact. Interviewees from the Non-UCLA-VFC Programs also commented on the value of taking an initial needs assessment. All participants suggested using ongoing programmatic evaluations for quality improvement purposes.

Interviewees recommended measuring food acceptability, behavioral changes, and clinical outcomes as key indicators of program success. Food acceptability refers to participants’ likeability of food choices. Examples shared included assessing patients’ receptiveness through “refill rates” (the percentage of patients returning to utilize the food program’s resources and offerings) and conducting phone calls to participants to collect feedback. Behavioral changes included an increased intake of fruit and vegetable servings. Clinical outcomes were the measured clinical biomarkers (e.g., hemoglobin A1c, blood pressure, weight). Interviewees unanimously emphasized the importance of gathering patient feedback on various program components to drive improvements.

Both the University-Affiliated Program and the Nonprofit Program inquired about food accessibility in their evaluations and considered patient perceptions and reactions to specific foods. By soliciting community input in their evaluations, these two programs also gained an understanding of the familiarity, cultural relevance, and acceptance of different foods or meals. Their interviews highlighted how multiple families changed their food preferences after one member of their family participated in the food program. Interviewees commented on the need to assess whether a prepared meal program adequately addresses food insecurity and whether the program results in lasting changes toward healthy-eating behaviors.

## 4. Discussion

The UCLA-VFC Food Program serves as a notable case study of how a university mobilized existing infrastructure and resources to address emergent community needs during the COVID-19 pandemic. This initiative advanced beyond handing out ingredients to distributing prepared meals, thus overcoming barriers such as time constraints and kitchen access. Similarly, community food programs in Detroit distributed meals during the pandemic, noting similar operational changes that allowed them to provide curbside “to-go meals” [17]. These programs consisted of previously established organizations which evolved to meet the needs of their community. In contrast, the UCLA-VFC Food Program was not originally designed as a long-term program. However, its short-term impact on participants highlighted potential considerations for prepared meals initiatives that might follow it. Reflections provided by program leaders and administrators highlighted four common takeaways: (1) the critical role of leadership and a collaborative culture; (2) the importance of community partnerships and health education; (3) challenges related to logistics, funding, and sustainability; and (4) the need for assessment and evaluation. The interview responses provided insights on the structural components of each program, challenges within the implementation process, and perceived impact from program leaders’ points of view. Commonalities among interview responses highlighted four key themes as essential considerations for future food programs.

The UCLA-VFC Food Program demonstrated how visionary leaders from the university, clinic, and community could identify a collaborative solution that would unite the missions of all three parties. This led to UCLA Dining Services pivoting from serving college students to preparing meals for the VFC patient population in a time of crisis [12]. The UCLA-VFC Food Program serves as an example of how community-focused, mission-driven organizations can unite diverse stakeholders around shared values.

Like many FIM pilot programs discussed in the literature, the UCLA-VFC Food Program incorporated elements of community partnership and health education [18]. However, the nature of partnerships across all the programs varied. The UCLA-VFC Food Program’s emphasis was more on emergency relief, while other programs were designed for long-term impact. These other programs provided a more robust emphasis on nutrition education by providing educational materials along with the food resources. For example, the University-Affiliated Program incorporated “meal idea cards” and medically tailored ingredients (e.g., low-sugar food boxes for participants with diabetes). This approach aligns with research suggesting that medically tailored meals can reduce healthcare costs by preventing chronic illnesses; however, such results are often limited by small study numbers and require further investigation [8,19].

The Nonprofit Program had the unique ability to serve as a consultant for other community organizations by guiding each of them to implement food programs within their existing infrastructures. This program underscored the importance of adaptability and innovation in addressing diverse needs. For example, one of the program’s partners had a medical assistant who first cooked sample meals for the patients, then provided ingredients and recipes so patients could make the same meals at home. Patients were also connected with a clinician and invited to meet with a health educator. A second affiliated program incorporated online cooking classes demonstrating ways to cook with a microwave or coffee pot for patients with limited access to fully stocked kitchens. By encouraging tailored approaches, the Nonprofit Program played a role in a variety of innovative and educational services. Lastly, the Public Health Program operated with a fruit and vegetable voucher that subsidized produce purchases at local grocery stores, which is similar to the produce-prescription model seen in other programs across the nation [10,20].

All three of the Non-UCLA-VFC Programs in this study provided raw materials and ingredients to their participants through food pharmacies or food pantries, while the UCLA-VFC Food Program provided fully prepared meals. Further research is needed to characterize the diverse ways health agencies and community organizations find synergy in their efforts to increase food access, health, and wellness. In terms of logistics and operations, both the UCLA-VFC Food Program and Non-UCLA-VFC Programs relied on similar funding sources: philanthropy, grants, and volunteers. However, the Nonprofit Program in its consulting role was able to scale its impact by supporting the growth of food initiatives across its network. By leveraging existing community assets, such as food voucher programs, nutrition education programs, and local housing resources, the Nonprofit Program demonstrated the power of collaboration to meet the community’s diverse needs. The Nonprofit Program also depended heavily on buy-in from university health systems and local health networks to support efforts over time. Interviewees across all programs in this study emphasized the importance of maintaining partnerships beyond local clinics to ensure adequate resources and personnel to support their work.

Sustainability remains a challenge across food programs, and future research is needed to explore additional funding avenues, such as partnerships with health insurance providers. A published survey reported that over 50% of US health plan leaders are interested in FIM programs as a method to decrease the need for GLP-1 agonists [10]. With the rising use and costs of these weight loss drugs to manage patients’ metabolic health, collaborations between providers, payers, and policymakers to invest in cost-saving, preventive FIM solutions may become more feasible.

Lastly, assessment and evaluation emerged as a critical component for programs. Although the UCLA-VFC Food Program did not implement formal evaluations, Non-UCLA-VFC Programs provided valuable insights into how structured feedback—such as food acceptability, behavior changes, and clinical outcomes—can guide the evolution of these initiatives. However, none of the programs fully implemented all of the suggested evaluation metrics, indicating a need for further research to establish best practices in program assessment. The improvement of food programs depends on the continued evaluation of their impacts on food insecurity, health behaviors, and cost savings.

A preliminary descriptive analysis of the UCLA-VFC Food Program suggests that adult participants maintained their blood pressure and weight one year after participating in the program, which is clinically relevant given that most middle-aged adults gain two pounds per year [21,22]. A future analysis should adjust for the baseline BMI and blood pressure measurements and track changes over time. Although other FIM approaches have also shown preliminary promise in addressing food insecurity and combatting downstream effects such as cardiovascular disease, diabetes, and obesity, the results are largely based on pilot programs and short-term evaluations [9,10,23]. Future research could also evaluate whether meal delivery programs are associated with fewer emergency department visits and lower medical costs [8,19]. Additionally, more research is needed to investigate best practices for implementing nutrition education. Considerations may include accounting for patients’ traditions, cultures, and values [24].

If further investigation demonstrates clinically beneficial outcomes and cost-savings, there may be reasonable rationale for health insurance companies to support FIM interventions. This could help provide a long-term solution to financing FIM programs, inform healthcare policy, and improve guidelines. There may also be opportunities to garner support from health insurers and policymakers to make FIM approaches an integral part of healthcare systems or spread similar programs into other underserved settings such as rural communities.

Overall, more rigorous evaluations are needed to understand the broader impact of FIM initiatives, inform the practice of preventive healthcare, and shape future collaborative models.

### Limitations

We acknowledge the study limitations particularly as they relate to the short duration of the UCLA-VFC Food Program and the limited use of evaluation tools to accurately measure the program’s qualitative impact. Therefore, reflections from interviewees may only reflect short-term impacts, leading to an incomplete understanding of the program’s long-term effect on the community. Findings from this emergency response may lack generalizability to other programs intended for long-term sustainability within an FQHC or another community health organization. Even so, the UCLA-VFC Food Program serves as a valuable proof of concept. The inclusion of Non-UCLA-VFC Programs in the interviews suggests that certain universal considerations apply to both short- and long-term programs.

Given the qualitative nature of the study, which relied on interviews and thematic analysis, there is potential for bias and subjectivity in interpreting the data. To mitigate this, we employed a rigorous process, including the development of a codebook and the use of a third reviewer to refine and validate themes iteratively. Nonetheless, future research could benefit from incorporating quantitative analyses and a mixed-methods approach to evaluate food programs and assess their immediate and long-term health impacts. Such an approach could provide robust data to support the scaling and dissemination of food programs, helping to ensure their sustainability and broader impact.

## 5. Conclusions

Our findings contribute valuable insights to the broader understanding of how prepared meal programs can mitigate food insecurity, particularly during crises like the COVID-19 pandemic. The UCLA-VFC Food Program specifically highlights the unique potential of university–community partnerships in addressing food insecurity, while our overall thematic analysis of interviews with FIM program leaders indicates that future iterations could be enhanced by integrating health education and literacy efforts, securing diversified funding to ensure sustainability and scalability, and establishing a robust evaluation framework. This work adds to the existing literature on food programs, including food pharmacies, meal delivery systems, and produce-prescription initiatives, offering a deeper understanding of how these approaches can address food insecurity on a larger scale.

In conclusion, this study offers preliminary evidence supporting the potential of university–community collaboration and similar partnerships to address food insecurity. Unlike previous research that emphasized clinical outcomes, our findings provide a contextualized understanding of programmatic implementation. While further evaluation is necessary, this work lays the groundwork for a collaborative model aimed at addressing social determinants of health.

## Figures and Tables

**Table 1:** Demographic Characteristics of UCLA-VFC Food Program Participants.

Variable	Category	N = 6088	Percentage
Age (years)	0–19	1917	31.49
	20–69	3792	62.29
	70+	367	6.03
	Unknown	12	0.20
Gender	Male	2282	37.48
	Female	3806	62.52
Ethnicity, Race	Indigenous	9	0.15
	Hispanic white	4641	76.23
	Non-Hisp white	505	8.30
	Non-Hisp black	195	3.20
	Non-Hisp Asian	115	1.89
	Unknown/mixed	623	10.23
Medical Insurance	Medicare	369	6.06
	Medi-Cal	2146	35.25
	HCLA	2836	46.58
	LA County	83	1.36
	Private Insurance	68	1.12
	Self-pay	474	7.79
	Other/Unknown	112	1.84
Housing	Shared Housing	64	1.05
	Not Homeless	5666	93.07
	Shelter	77	1.26
	Homeless	189	3.10
	Other/Unknown	92	1.51

**Table 2:** Clinical Trends of UCLA-VFC Food Program Participants. Clinical Results by Age Group (kids <20 yrs.; adults 20+ yrs.). Data are based on multiple encounters per individual.

Variable	Category	Children (<20 yrs.)					Adults (20+ yrs.)				
		Encounters (N)	Percent	Mean	Std. Dev.	*p*-Value	Encounters (N)	Percent	Mean	Std. Dev.	*p*-Value
Time period *	pre-interv	4279	36.55				15081	41.68			
	interv	3531	30.16				9453	26.13			
	post-interv	3898	33.29				11646	32.19			
Mean BMI	pre-interv	2650		21.49	6.08		13308		30.70	6.76	
	interv	1880		22.68	6.15		6893		30.71	7.47	
	post-interv	2875		22.30	6.67	0.0001	9242		31.14	7.09	0.0001
Mean systolic BP	pre-interv	2445		105.54	10.93		14618		124.56	19.55	
	interv	1841		107.84	11.15		7553		125.80	19.74	
	post-interv	2694		105.31	11.45	0.0001	13517		125.93	19.40	0.0001
Mean diastolic BP	pre-interv	2445		66.07	7.82		14618		74.29	9.92	
	interv	1841		67.34	7.82		7553		75.40	10.07	
	post-interv	2694		64.77	8.17	0.0001	10289		74.22	9.87	0.0001
Mean weight in lbs.	pre-interv	4178		63.51	55.41		17695		171.16	39.91	
	interv	3293		67.42	60.49		10679		171.23	41.28	
	post-interv	3678		61.66	61.66	0.0001	13350		172.72	40.25	0.0082

* Pre-intervention = 1 August 2019–31 July 2020. During intervention = 1 August 2020–31 July 2021. Post-intervention = 1 August 2021–31 July 2022.

**Table 3:** Program Details and Characteristics of Interviewees.

Description	Location	Facility	Strategy	Interviewee Role
**UCLA-VFC Food Program**				
University and local clinic	Los Angeles, CA	Venice Family Clinic	Fresh produce distribution, prepared meal program	Clinic CEO, Program Manager, Academic Executive, Clinicians
**Non-UCLA-VFC Programs**				
*University-Affiliated Program* Two university-affiliated clinics	Southwest Region, US	Two university-affiliated clinics	Food pharmacy, food banks, nutrition education	Program Director, Population Health Interventionist
*Public Health Program* A county-run program	Southern California, US	Community clinics, local grocery stores	Fresh produce prescription	Program Manager
*Nonprofit Program* Community clinics, food banks, and other FIM programs	Northern California, US	Nonprofit clinics	Food banks, farmer’s markets, grocery stores, food pharmacy	Associate Director

**Table 4:** Key Themes, Subthemes, and Representative Quotes from Key Informant Interviews.

**Theme 1: Leadership and Culture**			
**Visionary Leadership**	**Mission-Driven Ideology**	**Community-Building and Mobilizing Culture**	**Food is Medicine (FIM) Ideology**
“This is bringing healthy food into health care…food does matter, it matters so much that we’re creating space in the clinic for us to give it to you… I think [this] allows healthcare providers to think more holistically about patients [as] having legitimate basic needs that are impacting their health.” “How can you take the fact that your patient with diabetes has food insecurity and integrate that maybe into the medical care plan?” “Have an open mind, be creative and open to change.” “The incredible number of key stakeholders and the tentacles we have in our own…community creates these opportunities that leverage resources and bring people together to do extraordinary things.”			
**Theme 2: Partnerships and Health Education**			
**Partner with Organizations and Healthcare Teams**	**Partner with Families**		**Partner with Health Educators**
“Look at your strengths, who’s working on equity, justice, food, there’s some string of commonality among all those groups, and bring them together in that collective impact model and mobilize them…it’s a community organizing effort to bring people and build a common goal.” “…one of my first key learnings with Food Service Programs is you can’t have just one health care champion, you really need to invest heavily in having the entire health care team be bought in so that if you lose a singular champion, you’re not starting all over again.” “Creating a coalition within your community, whether it’s your clinic community, or broader community of people that bring different skills and have different stakes, and it would be critical to then learn how you could leverage resources and make sure you have common goals.” “…we try to supplement that with health education, so people start to understand what they’re eating and why it’s important.” “Know the community and ask before you come up with something that they would want.”			
**Theme 3: Logistics, Funding, and Sustainability**			
**Consider Household Size for Food Distribution**	**Minimize Barriers to Increase Accessibility**		**Embrace Philanthropy and Volunteerism**
“I do think that their approach of feeding the whole family was really good. Most food pharmacy programs don’t do that.” “We did have a few patients who needed the meals, but maybe didn’t have transportation.” “It was also nice because there were many younger people from a whole variety of communities coming out because they were accustomed to volunteering for different places, whether they were [college] students, or whether they were high school students, or whether they were just living in the neighborhoods.” “I think the other success was our ability to locate funds for it… It was an extraordinary community event in the sense that people who saw us, and saw it growing would call up and say, ‘How do I help you? What more do you need?’”			
**Theme 4: Assessment and Evaluation**			
**Identify and Adjust along the Way** **(What is Working/What is Not)**		**Address Feedback and Input from Families**	
“We had a few patients start picking up and then they just stopped…halfway through, or they came in a few times and then didn’t come back. And mostly because what I heard from some patients is like, ‘Oh, that’s not the type of food that we eat’. “I don’t know how the families felt about the meals… I don’t know… if they were meals that were culturally…recognizable, you know?” “Maybe what I would do differently is probably have feedback, like focus groups…and participation of families. Like, which meals were really tasty? Which ones…do less of? Is there anything you would like to see on the menu?” “The meals weren’t specifically tailored to that particular community. I would have really liked for us maybe to ask the patients… ‘What would you like to have?’ Or ‘What [are] some of the items that you want included?’”			

## Data Availability

The data presented in this study are available upon request from the corresponding author due to the small sample size and the associated risk of breaching participant confidentiality.

## Abbreviations

The following abbreviations are used in this manuscript:

EHR	Electronic Health Record
FIM	Food Is Medicine
FQHC	Federally Qualified Health Center
IT	Information Technology
Semel HCI	Semel Healthy Campus Initiative Center at UCLA
UCLA	University of California Los Angeles
UCLA-VFC Food Program	UCLA-VFC Emergency Prepared Meal Program
VFC	Venice Family Clinic
